# Phylogenetic analysis of prospective *M. bovis* antigens with the aim of developing candidate vaccines for bovine tuberculosis

**DOI:** 10.1186/s43141-023-00552-3

**Published:** 2023-10-12

**Authors:** Zhandos Abay, Sandugash Sadikalieva, Kamshat Shorayeva, Bolat Yespembetov, Makpal Sarmykova, Kuanish Jekebekov, Gaukhar Tokkarina, Zharkinay Absatova, Elina Kalimolda, Yeraly Shayakhmetov, Sabina Moldagulova, Aisha Issabek, Syrym Kopeyev, Alisher Omurtay, Kainar Barakbayev, Markhabat Kassenov, Nazym Syrym, Yergali Abduraimov, Kunsulu Zakarya, Ainur Nurpeisova

**Affiliations:** 1 Research Institute for Biological Safety Problems, Gvardeysky, 080409 Kazakhstan; 2 al-Farabi Kazakh National university, Almaty, Kazakhstan

**Keywords:** Bovine tuberculosis, Vaccine, *M. bovis*, ESAT-6, TB10.4, PCR, Sequencing, Senger

## Abstract

**Background:**

Bovine Tuberculosis is a respiratory disease caused by the pathogen *Mycobacterium bovis (M. bovis)* that infects cattle. Though rare, this disease can also affect humans, as well as domestic and wild animals, making it a serious concern. Therefore, searching for alternative and new vaccines with high efficiency and safety is the main goal in bovine tuberculosis prophylaxis. New vaccines, known as vector vaccines, have the potential to become safe and effective alternatives to the traditional BCG vaccine. In this study, two major immunodominant proteins of *M. bovis* Esat-6 and TB10.4 were utilized to create a vector vaccine for bovine tuberculosis.

**Methods:**

The Esat-6 and TB10.4 genes were amplified by PCR. The amplified and purified PCR products were sequenced by the Sanger method. Assembly and multiple alignments of amplicon nucleotides were carried out in the MEGA 11 software.

**Result:**

Two genes of the local strain *0078-M. bovis-8/RIBSP* were sequenced. The nucleotide sequences were deposited in the GenBank database. Comparative analysis of the nucleotide sequences of the ESAT-6 and TB10.4 genes established 100% identity of the compared strains of *Mycobacterium*.

**Conclusion:**

Through the use of phylogenetic analysis, it has been confirmed that the amplified genes are related to the mycobacteria genus. This discovery allows the development of a vector vaccine against bovine tuberculosis utilising these genes.

## Background

Bovine tuberculosis is a disease that can be passed from animals to humans under certain circumstances, such as drinking raw milk or inhaling aerosolized droplets or dust particles. The cause of this disease is *Mycobacterium bovis (M. bovis)* [1, 2], a type of mycobacterium that is closely related to *Mycobacterium tuberculosis (M. tuberculosis),* a group of mycobacterium species that cause human tuberculosis. These species are believed to have originated from *Mycobacterium canettii (M. canettii),* which was first identified in 1979 [3]. The group comprises seven related species adapted to various hosts, including *M. tuberculosis, M. bovis, M. africanum, M. microti, M. caprae, M. canettii,* and *M. pinnipedii.* These related species can be seen as a series of ecotypes adapted to a particular host, rather than species that form part of the strain line. A notable characteristic these ecotypes share is the absence of a chromosome site. *M. bovis* is the final ecotype in the evolutionary series related to *M. tuberculosis* [4–6].

Studies suggest that the critical immunodominant proteins *of M. bovis* are Esat-6 and TB10.4, which are utilized in producing modified vector BCG vaccines. These proteins are secreted by the type VII secretion export system, with Esat-6 on EsxA and TB10.4 on EsxH. The loss of RD1, an important region for virulence, is the primary attenuation factor in BCG generation [7–9]. ESAT-6, or Rv3875, is predominantly secreted during culture. *M. bovis* is genetically similar to *M. tuberculosis*, the primary cause of human tuberculosis, with a 99.95% nucleotide level identity [10]. The secretion of ESAT-6 contributes to numerous mycobacterial processes that promote pathogenesis, such as suppressing T-cell IFN-γ secretion and inducing the death of host cells. Additionally, it forms pores in mycobacteria-containing vacuoles' membranes, facilitating their entry into the cytosol by "slipping" from the infected host cell. ESAT-6 promotes granuloma formation by activating metalloproteinase-9 on adjacent epithelial cells [11, 12]. It is a potent T-cell antigen, and its immunological properties have been extensively studied in animal models. They are recognized as a visible target in the early stages of *M. tuberculosis* or *M. bovis* infections. While the EsxH system's role is not fully understood, it is known to participate in iron absorption. Recently, researchers discovered a zinc-bonding site in the TB10.4 protein, indicating its direct involvement in zinc absorption [13]. TB10.4 elicits both CD4 and CD8 responses in humans and mice and has been included in vaccines undergoing clinical trials. Despite their different functions in mycobacteria's growth and virulence, ESAT-6 and TB10.4 exhibit similar immunological characteristics and are well-recognized in infected laboratory animals and sick humans [14–16].

Currently, there is a significant focus on developing vector vaccines and vaccines based on nucleic acids due to their potential efficacy. Researchers have studied viral vectors such as adenoviruses, poxviruses, and vesicular stomatitis viruses (VSV) as delivery systems for bTB antigens. These vectors have proven effective in infecting cells and eliciting an immune response against target antigens. The main challenge in developing vector vaccines is selecting a safe and optimal vector and incorporating genes that will protect the recipient [17, 18]. The latest discovery in this field is the use of attenuated influenza viruses as vectors for delivering target bTB antigens.

Thus, this study aims to amplify the Esat-6 and TB10.4 genes of the local M. bovis strain and compare them phylogenetically to create effective vector vaccines against bovine tuberculosis.

## Methods

### Mycobacteria and DNA extraction

The *0078-M. bovis-8/NIIPBB* strain was received from the RIBSP collection of microorganisms. The *0078-M. bovis-8/RIBSP* strain was isolated from infected cattle through culturing in 2014. DNA extraction was performed using a QIAamp DNA Mini Kit (Qiagen) according to the manufacturer's instructions. DNA purity and quantity were verified using a mySPEC (VWR) spectrophotometer based on optical density at the wavelength of 260 nm and 280 nm (E260/E280).

### Genes amplification

To run PCR, specific primers for Esat-6 and TB10.4 were designed using the Primer-BLAST online software (http://www.ncbi.nlm.nih.gov/tools/primer-blast) to produce 285 to 280 b.p. sized amplicons (for ESAT-6 and TB10.4, respectively). Primers were synthesized on an automatic DNA/RNA oligonucleotide synthesizer (Synthesizer H-16, K&A Laborgeraete, Germany) as per manufacturer protocol [19]. Amplification of the Esat-6 and TB10.4 genes was using a reaction mixture in the volume of 25 μL: Taq DNA polymerase containing MgCl_2_ – 0.3 μL; 10 × PCR buffer – 2.5 μL; specific primers (20 pM) – 1 μL of each; dNTP mixture (10 mM) – 1 μL; isolated DNA – 5 μL; and deionized water – 14.2 μL. We amplified nucleotides using the MiniAmp Thermal Cycler (Applied Biosystems) under the following conditions: 94 °C – 5 min, 94 °C – 1 min, 55/62 °C – 40 s, 72 °C – 1.30 min (35 cycles), and 72 °C – 7 min, 4 °C – cooling and storage for an indefinite amount of time. The DNA amplicons underwent thorough examination using the horizontal electrophoresis equipment (Sub Sell GT, BioRad). The resulting agarose gel was captured by the Imaging System (GelDoc Go, BioRad) for analysis.

#### Determining and analyzing nucleotide sequences

Samples for sequencing were prepared using the method proposed by Ausubel et al. Following Senger’s method, the sequencing test was performed on a BigDye® Terminator v1.1 Cycle Sequencing Kit (Applied Biosystems, USA) as per the manufacturer's recommendations. As per the manufacturer's protocols, we sequenced DNA in an ABI PRISM 310 Genetic Analyzer (Applied Biosystems, USA). We read the electrophoresis images in the Sequencing Analysis Software (Applied Biosystems, USA). The assembly and multiple alignments of amplicon sequences and built phylogenetic trees, and analyzed nucleotide sequences were matches using the MEGA 11 software package. Comparative analysis was carried out using the BLAST software, and homologous sequences were searched in GenBank.

## Results

### Target gene amplification

Esat-6 and TB10.4 are widely used mycobacterium antigens in vector and DNA vaccines. To amplify these genes, specific primers were selected and synthesized. The characteristics of synthesized primers are shown in Table 1.

**Table 1:** Characteristics of the selected primers

Proteins	Genes	Function	Primer name	Primer sequences (5^/^-3^/^)	Amplicon size (b.p.)	Agarose gel Electropherogram
*TB10.4*	*esxH*	Early secretion protein	Tb10.4_FP	CAGATGTCGCAAATCACAACTACCCCG	280	
			Tb10.4_RP	CAGTTGGCGGCTTCGGCGTGTCG		
*Esat-6*	*esxA*	Early secretion protein	Esat-6_FP	TAGATGACAGAGCAGCAGTGGAATTTCGCGGGTA	285	
			Esat-6_RP	AAGGAATTCTGCGAACATCCCAGTGACGT		

**Fig. 1:**
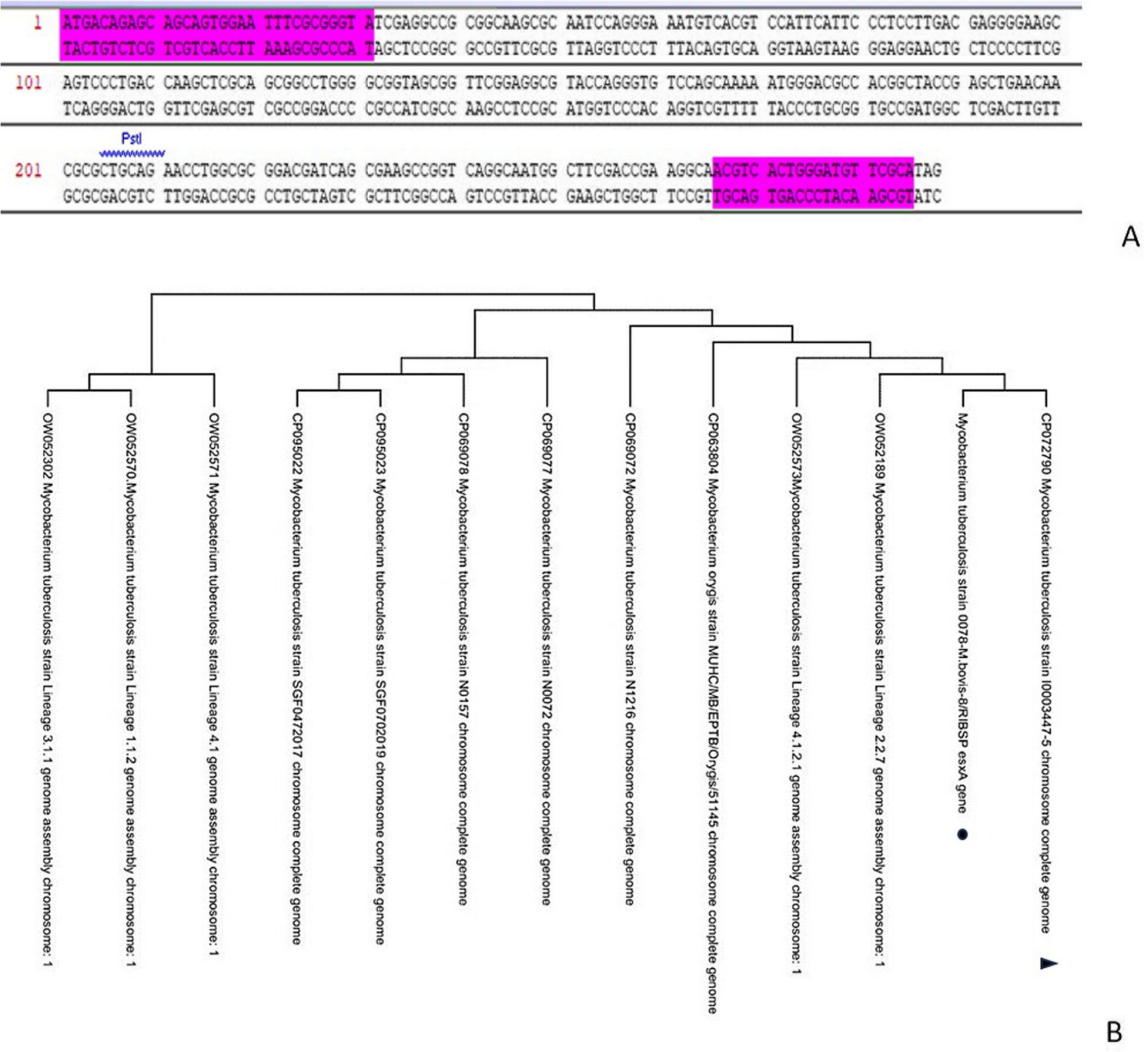
Phylogenetic analysis of the Esat-6 of the strain *0078-M. bovis-8/RIBSP*. **A** Sites of selected primers’ entry during Esat-6 gene amplification. **B** Phylogenetic tree for Esat-6 gene of the strain *0078-M. bovis-8/RIBSP*. Phylogeny of the Esat-6 gene was inferred using the maximum complex likelihood method in MEGA11. Studied sequence marked with black dot, referenced sequence marked with a black triangle

**Fig. 2:**
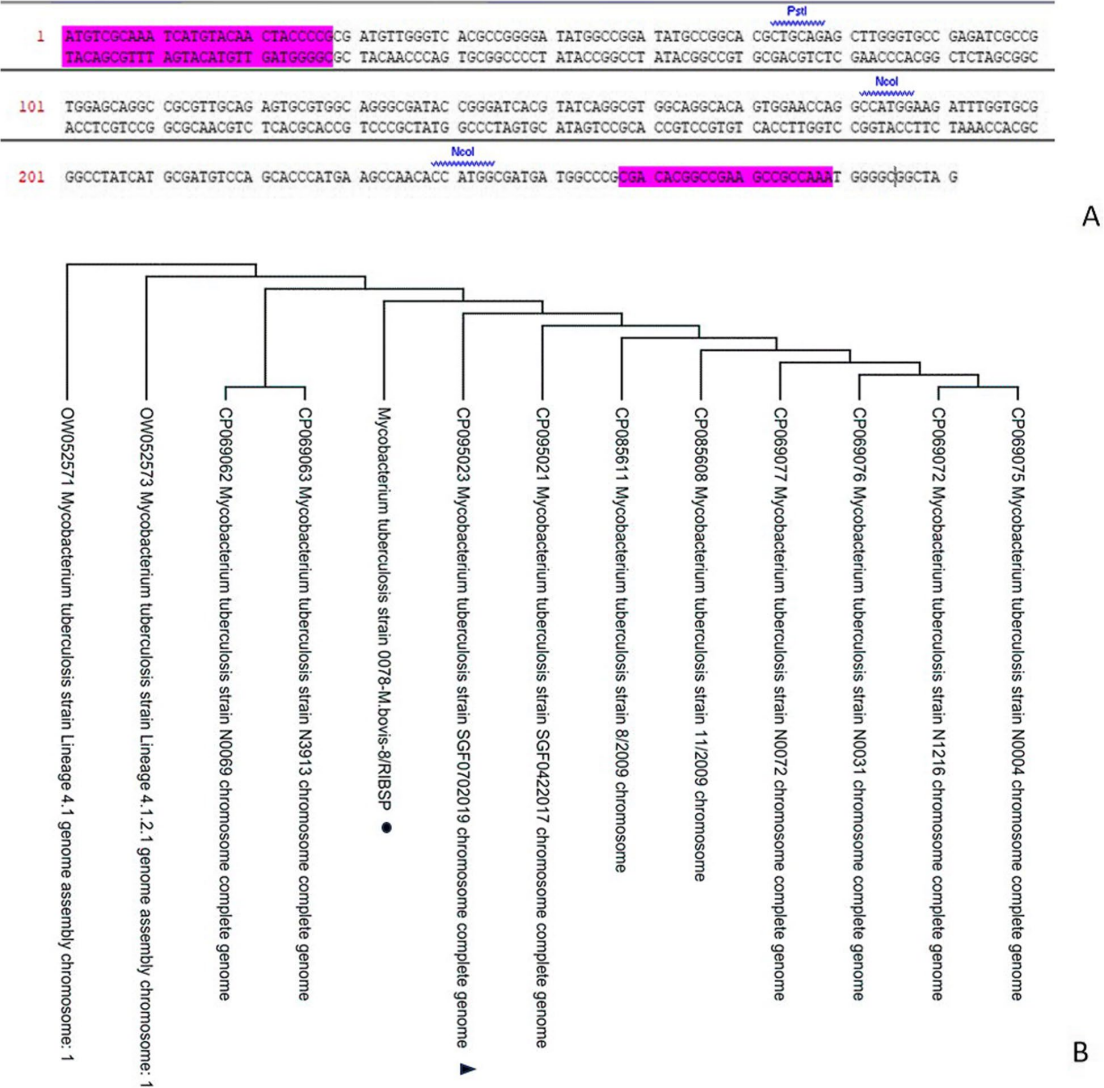
Phylogenetic analysis of the TB10.4 of the strain *0078-M. bovis-8/RIBSP*. **A** Sites of selected primers’ entry during TB10.4 gene amplification. **B** Phylogenetic tree for Esat-6 gene of the strain *0078-M. bovis-8/RIBSP*. Phylogeny of the TB10.4 gene was inferred using the maximum complex likelihood method in MEGA11. Studied sequence marked with black dot, referenced sequence marked with a black triangle

The constructed primers fully cover the nucleotide sequence of each gene—the TB10.4 and Esat-6 – and have corresponding molecular weights: 280 and 285 b.p., respectively. The selected primers’ entry sites are shown in Figs. 1 and 2. We used the primers to optimize our PCR procedures by trying various concentrations of some of the reaction mixture components because the target DNA's quality and quantity depend on the reaction mixture's proper component concentration and the right PCR temperature and time. Based on our work optimizing the time and temperature of PCR to amplify *M. bovis* genes, we selected the optimal annealing temperatures, which we established as 62 °C for Esat-6 and 55 °C for TB10.4.

### Comparative analysis of nucleotide sequences of the Esat-6 and Tb10.4 genes

After amplifying purified DNA of Esat-6 and TB10.4, sequencing was performed and obtained sequences were added to the GenBank database (ID OP610627, OP610628). We performed a phylogenetic analysis to establish the phylogenetic relationship of *M. bovis* based on the Esat-6 and TB10.4 genes isolated from an archived RIBSP *M. bovis-8* strain with similar gene samples from GenBank (Figs. 1 and 2).

In Fig. 1, we can see the nucleotide sequence of the *0078-M. bovis-8/RIBSP* strain’s Esat-6 gene belongs to the *Mycobacterium* family and forms a separate genetic branch with the I0003447-5 M*. tuberculosis* strain (CP072790) from GenBank.

Our comparative analysis of nucleotide sequences of the TB10.4 gene of the Kazakhstan *0078-M.bovis-8/RIBSP* strain with the most closely related strain from GenBank, namely, *M. tuberculosis* strain SGF0702019 (CP095023) has shown that the compared nucleotide sequences are 100% identical with no substitutions in the nucleotide sequence. It also provides evidence that the *0078-M. bovis-8/RIBSP* strain belongs to the *Mycobacterium* family and forms a separate genetic branch with the *M. tuberculosis* strain SGF0702019 (CP095023) from the GenBank database (Fig. 2).

## Discussion

The spread of *M. bovis* constitutes one of the highest-priority problems, which leads to large losses in agricultural animal populations [19].

The genomes of *M. bovis* and *M. tuberculosis* are more than 99.95% identical at the nucleotide level [20]. Based on the literature research, it has been discovered that mycobacterial proteins ESAT-6 (early secret antigen target-6) and TB10.4 *M.bovis* exhibit potential antigenic properties [10, 21, 22]. The ESAT-6 family comprises a group of known antigens that are affected by M. bovis deletions. The ESAT-6 protein was initially identified as a potent T-cell antigen that is secreted by *M. tuberculosis* [23]. Considering these characteristics, the TB10.4 and ESAT-6 proteins are promising antigens for developing candidate vaccines against bovine tuberculosis [22].

To develop a recombinant vaccine strain based on a reassortant influenza A virus expressing the secretory proteins Esat-6 and TB10.4 of the local strain of bovine tuberculosis 0078-*M. bovis*-8/RIBSP, we have amplified DNA of the ESAT-6 and TB10.4 gene regions of the Kazakh strain 0078-*M. bovis*-8/RIBSP. The resulting products were sequenced, and the resulting gene sequences were deposited in the GenBank database under ID numbers OP610627 and OP610628. A phylogenetic analysis of the ESAT-6 and TB10.4 genes was carried out to use them to create a vector vaccine against a strain of tuberculosis isolated in Kazakhstan. Our data show that, in terms of the ESAT-6 gene, it is closest to *0078-M. The Bovis-8/RIBSP* strain is *M. tuberculosis *I0003447-5 (ID: CP072790), isolated in the Jiangsu region, China, from a human skin biopsy in 2019. When aligning the nucleotide sequences of the two genes, there were no differences and no nucleotide substitutions. The similarity of the two sequences was 100%. According to the TB10.4 gene, the N0004_M strain is the closest. Tuberculosis (ID: CP069075) was isolated in India from human sputum in 2010 between the TB10.4 sequences and the reference strain N0004_M. tuberculosis (ID: CP069075), no substitutions and differences in nucleotides were detected, which shows 100% similarity of gene regions. Studies of closely related strains provide insight into the candidate genes or components that can be used in vaccine development. The study focused on a strain of bovine tuberculosis isolated in Kazakhstan. This regional feature is advantageous as it provides insight into the strains' genetic characteristics and relatedness in a particular geographic area, aiding disease development and control.

A limitation of the study is the sample size, meaning that only the ESAT-6 and TB10.4 genes of a single strain of *M. bovis* were sequenced in the study. The data obtained do not reflect the overall genetic diversity of *M. bovis* strains in Kazakhstan or other regions. Phylogenetic analysis was based on identifying the ESAT-6 and TB10.4 genes of the *M. bovis *strain with a limited number of reference strains [24]. A more extensive analysis using many reference strains in different geographic regions would better understand strain relationships and assignments. Findings may not apply to other TB strains or have broader implications beyond this strain detection [25].

To highlight these limitations, future studies will expand the sample size, including a broader range of reference strains, including whole-genome sequencing, and identify a comprehensive functional test to validate a vector vaccine against different tuberculosis strains.

Considering the promising results of live viral vectors, an alternative strategy for developing safe and effective vaccines for infectious diseases is using genetically modified vectors, i.e., non-pathogenic microorganisms (bacteria and viruses) that express the agent’s antigens [26–28]. The main challenge in building vector vaccines is to select an optimal and safe vector and insert genes that will protect the recipient [17].

The results of our study can be used in developing a new vector vaccine candidate for bovine tuberculosis through genetic manipulations, namely, reverse genetics of RNA-containing viruses capable of expressing immunodominant mycobacterial proteins Esat-6 and TB10.4 of the *M. bovis* strain.

## Conclusion

In this study, we acquired the nucleotide sequences of the Esat-6 and TB10.4 genes in the local *M. bovis *strain. Upon conducting a phylogenetic analysis of these genes, we found they exhibit 100% similarity with the mycobacteria genus. This discovery paves the way for utilising these genes to develop a vector vaccine against bovine tuberculosis.

## Data Availability

The sequenced sequence of two genes of the strain *0078-M.bovis-8/RIBSP* was published in GenBank under the following accession numbers: OP610627 and OP610628.

## References

[1] Palmer MV, Welsh MD, Hostetter JM (2012). Mycobacterial diseases of animals. Vet Med Int.

[2] Muller B, Durr S, Alonso S, Hattendorf J, Laisse CJM, Parsons SDC, van Helden PD, Zinsstag J (2013). Zoonotic Mycobacterium bovis-induced tuberculosis in humans. Emerg Infect Dis.

[3] Blouin Y, Hauck Y, Soler C, Fabre M, Vong R, Dehan C, Cazajous G, Massoure P-L, Kraemer P, Jenkins A, Garnotel E, Pourcel C, Vergnaud G (2012). Significance of the identification in the horn of Africa of an exceptionally deep branching Mycobacterium tuberculosis clade. PLoS One.

[4] Smith NH, Gordon SV, de la Rua-Domenech R, Clifton-Handley RS, Hewinson RG (2006). Bottlenecks and broomsticks: the molecular evolution of Mycobacterium bovis. Nat Rev Microbiol.

[5] Smith NH, Kremer K, Inwald J, Dale J, Driscoll JR, Gordon SV, van Soolingen D, Hewinson RG, Smith JM (2006). Ecotypes of the Mycobacterium tuberculosis complex. J Theor Biol.

[6] Smith NH, Hewinson RG, Kremer K, Brosch R, Gordon SV (2009). Myths and misconceptions: the origin and evolution of Mycobacterium tuberculosis. Nat Rev Microbiol.

[7] Ligon LS, Hayden JD, Braunstein M (2012). The ins and outs of Mycobacterium tuberculosis protein export. Tuberculosis.

[8] Pym AS, Brodin P, Brosch R, Huerre M, Cole ST (2002). Loss of RD1 contributed to the attenuation of the live tuberculosis vaccines Mycobacterium bovis BCG and Mycobacterium microti. Mol Microbiol.

[9] Lewis KN, Liao R, Guinn KM, Hickey MJ, Smith S, Behr MA, Sherman DR (2003). Deletion of RD1 from Mycobacterium tuberculosis mimics bacille Calmette-Guerin attenuation. J Infect Dis.

[10] Garnier T, Eiglmeier K, Camus JC, Medina N, Mansoor H, Pryor M, Duthoy S, Grondin S, Lacroix C, Monsempe C, Simon S, Harris B, Atkin R, Doggett J, Mayes R, Keating L, Wheeler PR, Parkhill J, Barrell BG, Cole ST, Gordon VS, Hewinson RG (2003). The complete genome sequence of Mycobacterium bovis. Proc Natl Acad Sci USA.

[11] Smith J, Manoranjan J, Pan M, Bohsali A, Xu J, Liu J, McDonald KL, Szyk A, LaRonde-LeBlanc N, Gao L-Y (2008). Evidence for pore formation in host cell membranes by ESX-1-secreted ESAT-6 and its role in Mycobacterium marinum escape from the vacuole. Infect Immun.

[12] Volkman HE, Pozos TC, Zheng J, Davis JM, Rawls JF, Ramakrishnan L (2009). Tuberculous granuloma induction via interaction of a bacterial secreted protein with host epithelium. Science.

[13] Andersen P, Andersen AB, Sørensen AL, Nagai S (1995) Recall of long-lived immunity to Mycobacterium tuberculosis infection in mice. J Immunol. 154(7):3359–72. 7897219

[14] Martinez-Olivares CE, Hernández-Pando R, Mixcoha E (2023). In silico EsxG EsxH rational epitope selection: Candidate epitopes for vaccine design against pulmonary tuberculosis. PLoS One.

[15] Lee YH, Hyun YS, Jo HA, Baek IC, Kim SM, Sohn HJ, Kim TG (2022). Comprehensive analysis of mycobacterium tuberculosis antigen-specific CD4+ T cell responses restricted by single HLA class II allotype in an individual. Front Immunol.

[16] Skjot RL, Brock I, Arend SM, Munk ME, Theisen M, Ottenhoff THM, Andersen P (2002). Epitope mapping of the immunodominant antigen TB10.4 and the two homologous proteins TB10.3 and TB12.9, which constitute a subfamily of the esat-6 gene family. Infect Immun.

[17] Dietrich J, Weldingh K, Andersen P (2006). Prospects for a novel vaccine against tuberculosis. Vet Microbiol.

[18] Draper SJ, Heeney JL (2010). Viruses as vaccine vectors for infectious diseases and cancer. Nat Rev Microbiol.

[19] Abitaev RT, Shoraeva KA, Nurpeisova AS (2021) Selection and synthesis of primers for generating genes encoding protective M. bovis proteins. Eur Sci Assoc 10(80):159–164.

[20] Mahendra P, Nigusa Z, Tanvir R (2014). Growing significance of Mycobacterium bovis in human health. Microbes Health.

[21] Whelan C, ShuralevE, O'Keeffe G, et al (2008) Multiplex immunoassay for serological diagnosis of Mycobacterium bovis infection in cattle. Clinic Vaccin Immunol. 15(12):1834–1838. 10.1128/CVI.00238-08 PMC2593169 18927068

[22] Skjot RLV, Brock I, Arend SM, et al (2002). Epitope mapping of the immunodominant antigen TB10.4 and the two homologous proteins TB10.3 and TB12.9, which constitute a subfamily of the esat-6 gene family. Infect Immun. 70:5446–5453. 10.1128/IAI.70.10.5446-5453.2002 PMC128304 12228269

[23] Shuqing L, Hong J, Shaohua H, et al (2014). Recombinant TB10.4 of Mycobacterium bovis induces cytokine production in RAW264.7 macrophages through activation of the MAPK and NF-κB pathways via TLR2. Mol Immunol. 62(1):227–34. 10.1016/j.molimm.2014.06.026 10.1016/j.molimm.2014.06.026 25019567

[24] Lawn SD (2011). Advances in tuberculosis diagnostics: The Xpert MTB/RIF assay and future prospects for a point-of-care test. Lancet Infect Dis.

[25] Thierry D (2019). Whole-genome sequencing of Mycobacterium tuberculosis: Current standards and open issues. Front Microbiol.

[26] Sorensen AL, Nagai S, Houen G (1995). Purification and characterization of a low-molecular-mass T-cell antigen secreted by Mycobacterium tuberculosis. Infect Immun.

[27] Pleschka S, Jaskunas R, Engelhardt OG (1996). A plasmid-based reverse genetics system for influenza A virus. J Virol.

[28] Guo J, Mondal M, Dongming Zh (2018). Development of novel vaccine vectors: Chimpanzee adenoviral vectors. Hum Vaccin Immunother.

